# Exploratory analysis of the key role of immune function changes in BPD

**DOI:** 10.1002/pdi3.2515

**Published:** 2024-12-21

**Authors:** Tianyi Wu, Xingmeng Fu, Xiaoxia Gong, Jingyi You, Zhou Fu, Chang Shu

**Affiliations:** ^1^ Department of Respiratory Children's Hospital of Chongqing Medical University Chongqing China; ^2^ National Clinical Research Center for Child Health and Disorders Chongqing China; ^3^ Ministry of Education Key Laboratory of Child Developmental and Disorders Chongqing China; ^4^ Chongqing Key Laboratory of Pediatrics Chongqing China

**Keywords:** BPD, differential gene analysis, enrichment analysis, GSEA analysis, immune function

## Abstract

Bronchopulmonary dysplasia (BPD) is one of the most prevalent and severe chronic lung diseases in premature infants. The objective of the current study was to screen for key BPD‐associated genes and pathways by transcriptomic analysis from clinical patients and animal models. In our study, the differentially expressed genes were screened from 58 children with 14‐day BPD and 40 normal children in the GSE32472 dataset of the Gene Expression Omnibus database. Then, we identified four hub genes (*Cd3e*, *Cd3g*, *Cd247*, and *Itk*) and a signaling pathway (T cell receptor signaling pathway) by Gene Ontology and Kyoto Encyclopedia of Genes and Genomes pathway enrichment analysis and protein‐protein interaction analysis. The differential expression of the relevant pathways and gene sets among the groups was verified via GSEA analysis. Subsequently, a rat model of BPD with hyperoxia‐induced lung injury was established, and the transcriptome sequencing of the whole lung tissue was performed. A similar analysis was done on the sequencing data of the hub genes and associated pathway screening to verify the accuracy. Ultimately, quantitative polymerase chain reaction was performed to validate the transcriptomics data of core gene expression in the rat model. Our study revealed that the downregulation of the expression of the above four key genes in the course of BPD leads to a decrease in the function of T cell receptor signaling pathways, it causes immune dysfunction and increases the severity of lung inflammation as well as susceptibility to other respiratory infectious diseases.

## Graphical abstract

Screening of key pathways and genes in the course of BPD using bioinformatics methods

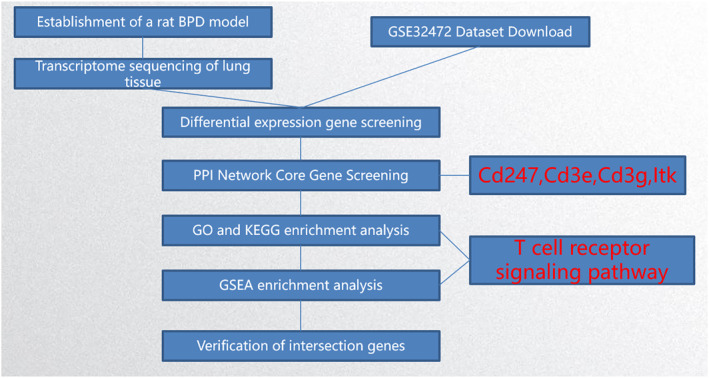

## 1 INTRODUCTION

Bronchopulmonary dysplasia (BPD) is one of the most prevalent morbidities associated with prematurity and the most common form of chronic lung disease in newborns, attributable to a multitude of factors. The documented predominant causes of death in children with BPD involve recurrent lower respiratory tract infections, persistent pulmonary hypertension, pulmonary heart disease, and sudden death.^1^ Previous studies have shown a high incidence of respiratory diseases and readmission rates in premature infants with BPD within 2 years of birth.^2^ A previous study from our group revealed that 121 children (<2 years old) with BPD were readmitted to the hospital 242 times for lower respiratory tract infections. Recurrent respiratory infections are extremely frequent in the early years of children with BPD, and while symptoms would alleviate with age, they are still more common in early‐term babies than in full‐term.^3^ Subjects with BPD exhibit a pronounced airflow limitation during adolescence and adulthood and may experience a severe decline in lung function during adulthood. Some studies report that BPD may be implicated in the development of chronic obstructive pulmonary disease in adulthood.^4^

The pathogenesis of BPD involves multiple prenatal and postnatal mechanisms which impair lung maturation, like abnormal repair responses to lung damage. These abnormal repair reactions are responsible for altered lung morphology, disruption of capillary gas exchange in the alveoli, and the pathological changes and clinical features of BPD.^5^ Overall, the pathogenesis of BPD remains elusive, with prematurity, genetic susceptibility, oxidative stress, stress damage, and infection being the predominant mechanisms.^6^, ^7^

At present, available therapies for BPD include anti‐inflammatory treatments such as glucocorticoids and inhaled nitric oxide (iNO), modifications to ventilation, lung surface‐active substances, early caffeine treatment, vitamin A, fluid restriction, antioxidants, and mesenchymal stem cell therapy. Nevertheless, no definite and safe targeted therapies are available for the prevention and treatment of BPD due to a poor understanding of the BPD mechanisms. None of the available therapies can cure BPD at the advanced stage.^8^ Therefore, the prevention and treatment of BPD have emerged as a priority, and it is a matter of urgency to find effective solutions to BPD symptoms.

Along with the recent advances in microarray technology, the availability of BPD datasets has provided a new boost to the study of its pathogenesis, and the screening for predictive biomarkers is expected to facilitate early diagnosis and targeted treatment. BPD‐related marker genes have been screened from the clinical datasets or animal models. There is a study that screened the differentially expressed genes (DEGs) of BPD and non‐BPD patients through the data set GSE108754 in the Gene Expression Omnibus (GEO) database, and carried out enrichment analysis and protein‐protein interaction (PPI) network analysis. Then, it was proved that *CD74* was a new predictor of BPD in premature infants using the method of quantitative polymerase chain reaction (QPCR).^9^ Another study examined the overall transcriptome changes of resident pulmonary immune cells by using a double hit model of clinically relevant BPD induced by prenatal chorioamnionitis and postpartum hyperoxia. The results of this study also proved that the expression of genes involved in various T lymphocyte functions was significantly down regulated in the course of BPD, especially the genes involved in T cell receptor signaling pathway.^10^ In addition, studies have also proved that *miR34a‐TNIP2‐IL‐1 β* Pathway plays a key role in the occurrence and development of BPD.^11^ In addition, some BPD related mutant genes^12^ have been found by sequencing the exons of BPD and control groups, but the results vary owing to the large individual variation in clinical study and the limitations of analyzing data from a single animal model.^6^, ^13^

Therefore, to explore the mechanisms of BPD development, we innovatively combined pre‐clinical data obtained from animal models of BPD and clinical data obtained from children with BPD in the GEO database for transcriptome analysis. The screening of hub genes and key pathways from the GEO database of children with BPD was performed and validated by the analysis of whole‐lung transcriptome sequencing data obtained from a rat model of BPD with hyperoxia‐induced lung injury in the hope of providing new potential targets for the early prediction and treatment of BPD.

## 2 MATERIALS AND METHODS

### 2.1 Dataset selection

We first selected the GSE32472 dataset from the GEO database. GSE32472 included 120 premature infants with gestational age <32 weeks, weight <1500 g and later diagnosed as BPD. Peripheral blood samples were collected on the 5th, 14th and 28th days after birth for full gene expression profile analysis, and were divided into time A, time B, and time C groups according to the sampling time. We selected 14 days of transcriptome data for bioinformatics analysis.

### 2.2 Animal models

Neonatal rat model of BPD with hyperoxia‐induced lung injury: pregnant Sprague‐Dawley (SD) rats were purchased from the Animal Experiment Center of Chongqing Medical University (CHCMU‐IACUC20220629003). SD neonatal rats born within 48 h were randomly divided into two groups (six rats per group), BPD group housed in a plexiglass oxygen chamber with continuous oxygen input for 14 days, and monitored using a digital oximeter to ensure the maintenance of 75%–85% oxygen concentration. The ambient temperature and humidity were maintained at 21–25°C and 60%–70%, respectively. The control group was placed in a normal environment. Every 2 days, the female rats in the BPD and normal groups were exchanged, supplied with water and feed, and the bedding changed.

Lung tissue sample extraction: rats were anesthetized by intraperitoneal injection of 10% chloral hydrate (3 μL/g) and sacrificed via cervical dislocation on day 14 of treatment. The lung tissue and bronchoalveolar lavage fluid samples were collected in EP tubes and stored in liquid nitrogen.

### 2.3 Transcriptome profiling via RNA‐seq

Total RNA was isolated and purified using the TRIzol method (Invitrogen) according to the manufacturer's protocol. The fragmented RNA was converted into cDNA with reverse transcriptase (Invitrogen SuperScript™ II Reverse Transcriptase, Item No.: 1896649). DNA‐RNA hybrid duplexes were then converted into DNA duplexes using *E. coli* DNA polymerase I (NEB, Item m0209) with RNase H (NEB, Item m0297) and dUTP solution (Thermo Fisher, item R0133). An A base was added to each end of the strand for its ligation to the terminal T base, and the fragments were screened and purified using magnetic beads. The second strand was digested with UDG enzyme (NEB, Item no. m0280) and amplified by PCR (pre‐denaturing at 95°C for 3 min, 8 cycles of denaturing at 98°C 15 s, annealing at 60°C for 15 s, extension at 72°C for 30 s, and final extension at 72°C for 5 min) to keep fragment size of 300 bp ± 50 bp in the library. Finally, we performed double‐end sequencing using Illumina Novaseq™ 6000 (LC‐Bio Technologies Co., Ltd.) in PE150 sequencing mode.

### 2.4 Screening for differential genes

Using the R package limma (version 4.2.1), differential gene expression in samples were analyzed, and the absolute value of log2FC ≥ 0.5 and *p* value < 0.05 were considered statistically significant.

### 2.5 Construction of PPI network and selection of core genes

The differentially expressed genes were analyzed by String (https://string‐db.org/), and a PPI network (PPI, interaction score >0.900) was constructed. The hub genes were detected using the CytoHubba plugin in Cytoscape software, and the core genes were screened.

### 2.6 GO and KEGG pathway analysis

Gene Ontology (GO) and Kyoto Encyclopedia of Genes and Genomes (KEGG) pathway enrichment analysis were performed on differentially expressed genes using the R package, clusterProfiler (version 4.2.1). *p* value < 0.05 was considered statistically significant.

### 2.7 GSEA analysis

GSEA software was used to analyze the significant difference in the expression of enriched genes between the BPD and normal groups (|NES| > 1, NOM *p*‐value < 0.05, FDR *q*‐value < 0.25).

### 2.8 Marker genes validation

Total RNA was extracted using the TRIzol method (Roche), followed by reverse transcription using EvoScript Universal cDNA Master (Roche) and amplification via qPCR using FastStart Essential DNA Green Master (Roche).

### 2.9 Western blot

Under the low temperature condition with ice, 250 uL of the whole protein extraction reagent system (KGP2100) was added, and the lung tissue was cut into pieces and placed in a homogenizer. After tissue homogenate, transfer the tissue homogenate into the precooled EP tube, and centrifuge at 4°C for 15 min at 12,000 × g. Transfer 200 uL of supernatant to a new precooled EP tube, add 50 uL of SDS‐PAGE protein loading buffer, shake and centrifuge, boil at 100°C for 5 min, and store in a refrigerator at 80°C. In addition, take the supernatant of residual tissue homogenate and use BCA protein content detection kit (KGPBCA) for BCA determination. The protein (30 μg) was separated on 12.5% SDS‐PAGE gel (YASE PG113) and transferred to PVDF membrane (Millipore). Place the membrane in a sealing solution (Biyuntian P0239) and incubate it at room temperature for 10 min. The transfer membrane was stained with the following primary antibodies: anti‐Itk (1:2000, Sanying Biology 21525‐1‐AP), anti‐Cd3e (1:500, Hua'an Biology HA500344), anti‐Cd3g (1:1000, Affinity DF8098), anti‐Cd247 (1:1000, Affinity DF6788), anti β‐Actin (1:5000, positive organism 700,068), incubated overnight at 4°C. Then, the horseradish peroxide coupled with the secondary antibody (1:5000, ABclonal AS014) was incubated at room temperature for 1 h. Use hypersensitive chemiluminescence reagent (Positive‐biological 17047) to develop in the chemical touch developer.

### 2.10 Statistical analysis

**FIGURE 1:**
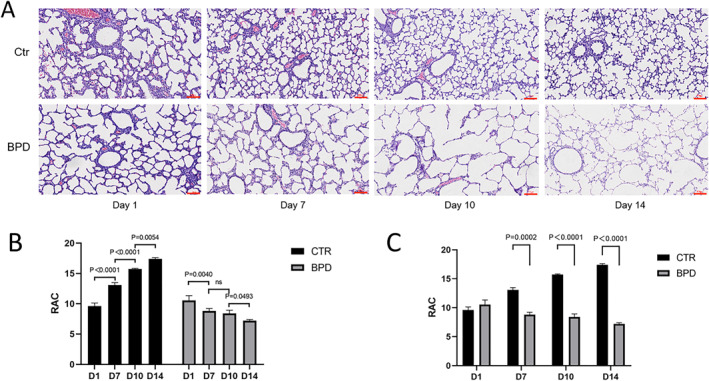
Structure of rat BPD lungs. (A) The sections of rat lungs, at day 1, 7, 10, and 14, exposed to room air (Ctr) or oxygen (BPD), were stained for H & E. The upper and lower panels are the control and the BPD group, respectively. Scale bars present 10X. As time passed, the lung tissue morphology of the control group became increasingly mature, the number and volume of alveoli gradually increased, the thickness of the pulmonary septum gradually decreased, the secondary septum gradually increased, and the alveolar ridge structure became more abundant, showing a gradual development. While the number of alveoli decreases, the volume increases, the alveolar ridges and secondary septa are not obvious or even disappear, and the number of pulmonary microvasculature decreases in the BPD group. (B) We can see that the RAC value of the control group gradually increased, indicating that the number of alveoli in the control group gradually increased and the development of lung tissue gradually improved. However, the RAC value of the BPD group was significantly lower than that of the control group (*p* < 0.01) and showed a downward trend, indicating a decrease in the number of alveoli and abnormal lung tissue development in the BPD group. (C) Compared with the control group at the same time point, the BPD group began to show significant changes from day 7 onwards. The RAC value of the BPD group was significantly lower than that of the control group (*p* < 0.01). BPD, bronchopulmonary dysplasia; RAC, radial alveolar count.

Statistical analysis was performed using GraphPad Prism 8 software. Data are presented as mean ± standard deviation (SD). Student's *T* test was used to compare two groups. And One‐way ANOVA was used to compare multiple groups of data in Figure 1B.

## 3 RESULTS

### 3.1 BPD was developed in neonatal rats exposed hypoxia

We performed HE staining on lung tissue sections of control group and BPD group rats on days 1, 7, 10, and 14 (Figure 1). As time passed, the lung tissue morphology of the control group became increasingly mature, the number and volume of alveoli gradually increased, the thickness of the pulmonary septum gradually decreased, the secondary septum gradually increased, and the alveolar ridge structure became more abundant, showing a gradual development. While the number of alveoli decreases, the volume increases, the alveolar ridges and secondary septa are not obvious or even disappear, and the number of pulmonary microvasculature decreases in the BPD group. Draw a vertical line from the center of the respiratory bronchioles to the nearest pleura or fibrous septum, and the alveolar count on this line is defined as the radial alveolar count (RAC). Observe the lung tissue structure of HE stained sections under a 100x magnification microscope. Extract five field counts from each section and take the average as the RAC.

### 3.2 *Cd3e*, *Cd3g*, *Cd247*, and *Itk* are the core genes in the progression of BPD

**FIGURE 2:**
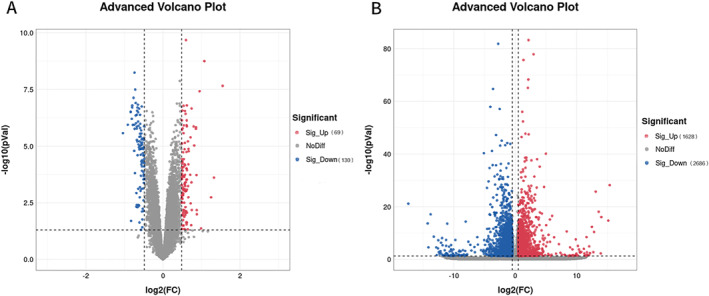
Gene expression profile in children with BPD. Volcano Map analyzed genes expression profile from (A). GSE32472 dataset of 14‐day postnatal children without and with BPD (Crt, BPD, respectively). (BPD *n* = 58, Ctr *n* = 36). (B) Rats, at day 14, exposed to room air (Ctr) or oxygen (BPD), (*n* = 6 for each group). Absolute value of log2(FC) ≥ 0.5, *p* < 0.05 were screened. Dots in blue and in red present down‐ or up‐regulated genes, respectively, while dots in gray present log2(FC) < 0.05 or *p* ≥ 0.05. BPD, bronchopulmonary dysplasia.

Gene expression profile data of 14‐day postnatal children with BPD (*n* = 58) and children free of BPD (*n* = 36) were selected from the GSE32472 dataset, and 199 differentially expressed genes (absolute value of log2[FC] ≥ 0.5, *p* < 0.05) were screened. Among them 130 genes were downregulated and 69 genes were upregulated. The transcriptome data of BPD rats (*n* = 6) and control rats (*n* = 6) were screened for 4314 differentially expressed genes. Among them 2686 genes were downregulated and 1628 genes were upregulated (Figure 2).

**FIGURE 3:**
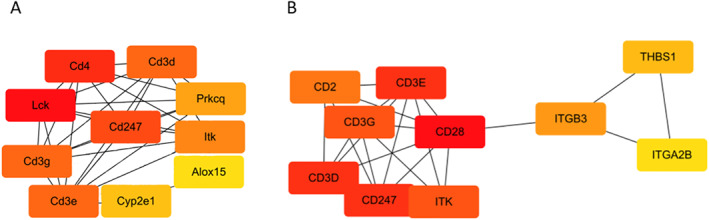
Protein interaction diagram of hub genes. (A) Hub genes of GSE32472 dataset. (B) Hub genes of Rats dataset. The connections between various genes indicate that they have interactions in biology. The connections between various genes indicate that they have interactions with each other.

Differentially expressed genes with adjusted *p*‐value < 0.01 were subjected to enrichment analysis, and genes without GO and KEGG annotations were excluded. The annotated genes were used to create PPI network graphs (interaction score >0.900) by String analysis. The hub genes were detected using the CytoHubba plugin in Cytoscape software, and the core genes were screened. However, due to the large pixel size of the original PPI network image, we have uploaded it to the additional file set. We believe that the common parts among the core genes identified in the rat model and database data analysis are the core genes that require more attention from us, as this proves that they play important roles in the BPD process of different species. Therefore, we will focus our attention on the core genes at the intersection of the two sets of data: *Cd3e*, *Cd3g*, *Cd247*, and *Itk* (Figure 3).

### 3.3 The results of GO and KEGG enrichment analysis showed significant enrichment of hub genes in the T cell receptor signaling pathway

The hub genes screened by CytoHubba were subjected to GO and KEGG pathway enrichment analysis.

**FIGURE 4:**
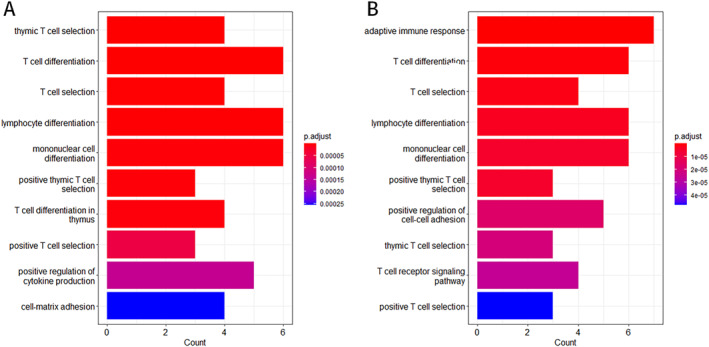
The results of GO enrichment analysis indicate the enrichment of core genes in T cell immune related biological functions. (A) GO enrichment analysis of GSE32472 dataset. (B) GO enrichment analysis of Rats dataset. The *X*‐axis represents the number of core genes included in this pathway. GO, gene ontology.

GO enrichment analysis revealed that the biological processes co‐enriched by both groups of hub genes mainly involved T cell differentiation, T cell selection, lymphocyte differentiation, monocyte differentiation, and thymic T cell selection (Figure 4).

**FIGURE 5:**
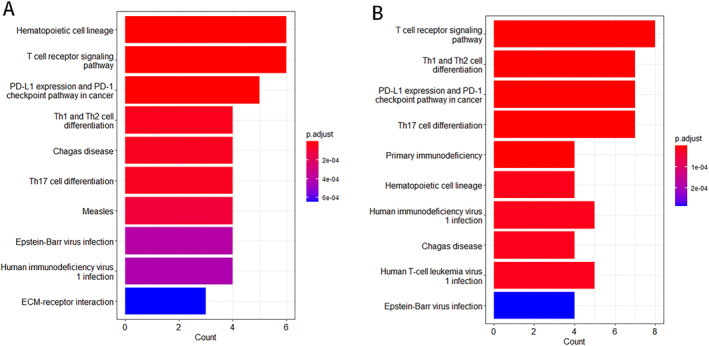
The results of KEGG enrichment analysis indicate the enrichment of core genes in T cell immune related pathways. (A) KEGG pathway enrichment analysis of GSE32472 dataset. (B) KEGG pathway enrichment analysis of rats dataset. The *X*‐axis represents the number of core genes included in this pathway. KEGG, Kyoto encyclopedia of genes and genomes.

KEGG pathway enrichment analysis disclosed that the main pathways co‐enriched by both groups of Hub genes included T cell receptor signaling pathway, *PD‐L1* expression, and *PD‐1* checkpoint pathway in cancer, Th1 and Th2 cell differentiation, Chagas disease, Th17 cell differentiation, epstein‐barr virus (EBV) infection, human immunodeficiency virus 1 infection, and other metabolic pathways (Figure 5).

The enrichment analysis showed that T cell‐related immune function plays an important role in the course of BPD.

### 3.4 The T cell receptor signaling pathway plays an important role in the progression of BPD

Following the exclusion of other viral infection‐related pathways, it was imperative to verify whether the expression of enriched hub genes was significantly different in the BPD and non‐BPD groups (|NES| (Normalized Enrichment score) > 1, NOM *p*‐value < 0.05, FDR *q*‐value < 0.25).

**FIGURE 6:**
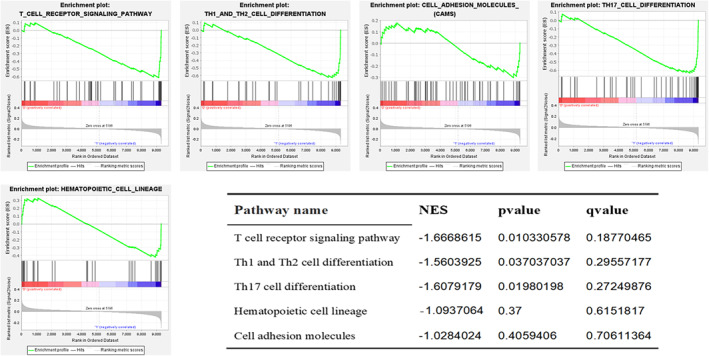
GSEA enrichment analysis report with statistical differences in the GSE32472 dataset. The horizontal axis represents each gene under this pathway, and the vertical axis represents the corresponding ES value. There is a peak in the line graph, which is the ES of this gene set. The genes before the peak are the core genes under this gene set. When the ES values are all negative, the corresponding gene to the right of its peak is the core gene in the gene set and is low expressed in the group. We can see that the differentially expressed pathways are all low expressed in the process of BPD. BPD, bronchopulmonary dysplasia; ES, enrichment score; NES, normalized enrichment score.

Among the differentially expressed hub genes and enriched pathways of the GSE32472 dataset in children with BPD and without BPD, the gene set of the T‐cell receptor signaling pathway was found to be significantly different (Figure 6).

**FIGURE 7:**
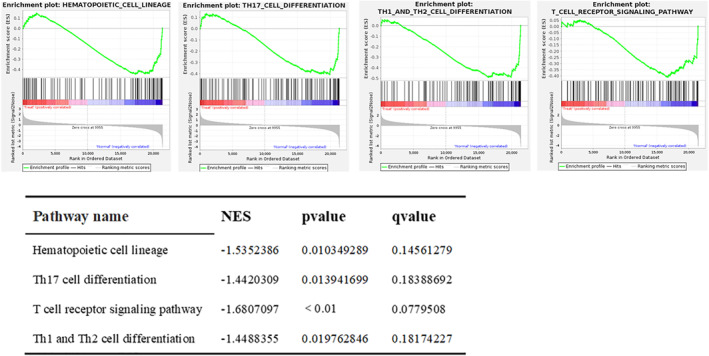
GSEA enrichment analysis report with statistical differences in the Rats dataset. The horizontal axis represents each gene under this pathway, and the vertical axis represents the corresponding ES value. There is a peak in the line graph, which is the ES of this gene set. The genes before the peak are the core genes under this gene set. When the ES values are all negative, the corresponding gene to the right of its peak is the core gene in the gene set and is low expressed in the group. We can see that the differentially expressed pathways are also all low expressed in the process of BPD. In addition, we can see that T‐cell receptor signaling pathways have the highest |NES| value, indicating the most significant difference in this pathway between the BPD group and the control group. BPD, bronchopulmonary dysplasia; ES, enrichment score; NES, normalized enrichment score.

GSEA analysis identified that the significantly different gene sets of the rats dataset in the BPD and normal groups were related to the following pathways: hematopoietic cell lineage, Th17 cell differentiation, T cell receptor signaling pathway, and Th1 and Th2 cell differentiation (Figure 7).

To sum up, GSEA analysis showed the significant downregulation of a metabolic pathway, the T‐cell receptor signaling pathway, in both the hyperoxia‐induced lung injury BPD rat model as well as the GSE32472 dataset. This indicates the important role of this pathway in the progression of BPD.

### 3.5 Different expression changes of hub genes in BPD rat lungs

**FIGURE 8:**
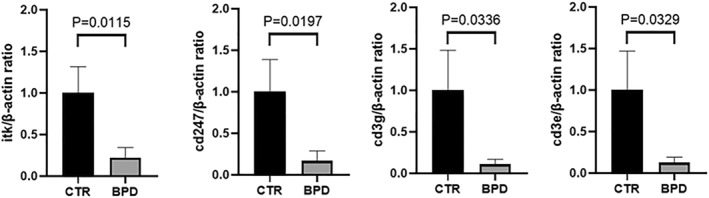
Down‐regulation of hub genes in BPD rat lungs. Total RNA was extracted from rat lungs and q‐RTPCR was performed. Quantitation of RT‐PCR for expression ratio of *Itk*, *Cd3g*, *Cd247* and *Cd3e* to β‐actin. Data present Mean ± SD and *t*‐test were performed. BPD vs. control group. The RT‐PCR results from four hub genes. There are significant differences in the expression of four hub genes at the DNA level. *n* = 4–5 for each group, *p* < 0.05. BPD, bronchopulmonary dysplasia.

The qPCR confirmation and validation of *Cd247*, *Itk*, *Cd3e*, and *Cd3g* gene expressions in lung tissues were done by replicating experiments in animal models (Figure 8). The results of qPCR showed that these four common differentially expressed genes had significant differences in the level of gene expression in the process of BPD, and the trend was consistent with the results of bioinformatics analysis.

**FIGURE 9:**
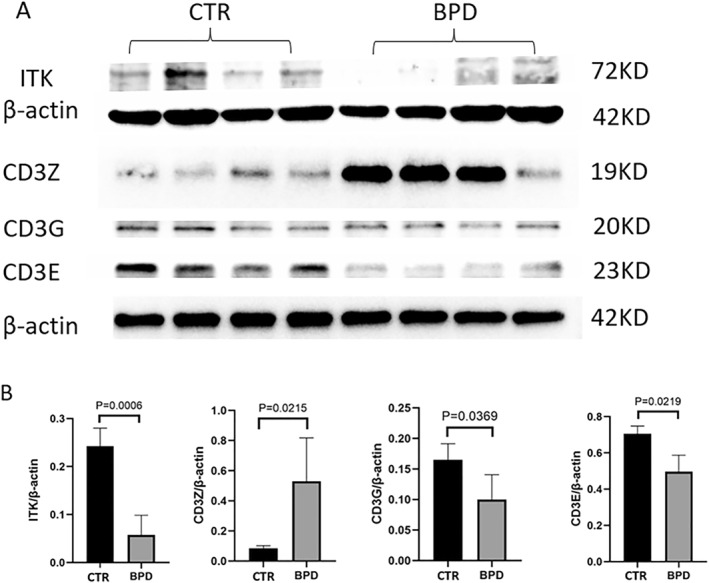
Down‐regulation of hub protein in BPD rat lungs. Total protein was extracted from rat lungs and Western Blot was performed. (A) Quantitation of Western Blot for expression ratio of *Itk*, *Cd3g*, *Cd247*, and *Cd3e* to β‐actin. (B) Data present Mean ± SD and *t*‐test were performed (*n* = 4–5 for each group, *p* < 0.05). BPD, bronchopulmonary dysplasia.

The Western Blot confirmation and validation of *Cd247*, *Itk*, *Cd3e*, and *Cd3g* protein expressions in lung tissues were done by replicating experiments in animal models (Figure 9). The Western Blot results showed that the protein expression of *Cd247*, *Itk*, *Cd3e*, and *Cd3g* was significantly different between the two groups. The protein expression difference trend of *Itk*, *Cd3e*, and *Cd3g* was consistent with the results of bioinformatics analysis and qPCR, while the expression difference trend of *Cd247* was opposite.

## 4 DISCUSSION

In this study, a rat model of BPD was established to detect transcriptomic differences in lung tissue during BPD disease. The core differential genes were first screened via the PPI network, and the enrichment analysis yielded BPD‐relevant biological functions and signal pathways. Meanwhile, GSEA analysis revealed that the expression of these gene sets in biological functions and signaling pathways indeed undergoes remarkable changes at the onset of BPD. Subsequently, the gene expression analysis of the GEO profile dataset provided further validation of the core genes screening. Eventually, the core genes *Cd247*, *Itk*, *Cd3e*, and *Cd3g* were screened for intersection in rat models and children with BPD. All four differentially expressed genes were found to be down‐regulated and strongly correlated with the enriched gene set of the T lymphocyte function pathway. Collectively, the downregulation of genes related to T lymphocyte function in the course of BPD is consistent with previous reports and merits our attention.^5^, ^6^

In the GSEA analysis of the rat model, we analyzed the gene set of the KEGG pathway for differential expression in the BPD group and the normal group. It was demonstrated that gene expression of the T cell receptor signaling pathway was markedly downregulated and further validated by data analysis of the GSE32472 dataset. Hence, the T‐cell receptor signaling pathway and its enriched genes warranted our concern. It has been documented that the inflammatory response plays a role in the progression of BPD.^10^, ^14^, ^15^ The dysregulation of immune function influences the development of inflammatory response, which is in line with our results.

*Cd247*, *Cd3e*, and *Cd3g* are located on the same cluster of chromosome 11, and together with *Cd3d* and the T‐cell receptor *α*/*β* and *γ*/*δ* heterodimers, they form the T‐cell receptor‐CD3 complex.^16^ In the T cell receptor signaling pathway, the TCR complex selects different classes of antigenic major histocompatibility complex (MHC) peptide complexes via *Cd8* and *Cd4* molecules. The interaction of the TCR with MHC peptides allows biological information to be transmitted through the cell membrane of T cells, thereby affecting the expression of various immune‐related genes.^15^ Hence, T‐cell signaling is critical for T‐cell proliferation and differentiation as well as immune tolerance, and its dysregulation may trigger severe autoimmune disorders.^17^

*Itk* is one of the key regulators of the T‐cell receptor signaling pathway and a member of the TEC non‐receptor tyrosine kinase family.^18^ It is involved in the downstream signaling of T‐cell surface receptors and is an important mediator of intracellular signaling in natural killer (NK) cells.^19^, ^20^, ^21^, ^22^, ^23^, ^24^ *Itk* has been reported as one of the key genes involved in the proliferation and differentiation of Th17 cells.^18^, ^25^ *Itk* is also essential for inflammation and tumorigenesis, and its deficiency induces severe EBV infections and diseases such as Hodgkin's and non‐Hodgkin's lymphoma, lymphoproliferative diseases, and mononucleosis.^26^, ^27^

The results of qPCR showed that at the level of gene expression, the differential expression trend of four core genes in BPD group and control group was down‐regulation, which indicated that in the course of BPD, the overall expression of TCR complex composed of *Cd247*, *Cd3e*, and *Cd3g* decreased, resulting in the abnormality of biological information transmission in the upstream of T cell receptor signal pathway, which led to the expression of downstream immune related genes, causing immune dysfunction. As a key gene involved in downstream signal transduction of T cell receptor signal pathway, the decrease of *Itk* expression further leads to abnormal immune function. The results of WB revealed that the protein expression of *Cd247* was contrary to that of the other three genes. *Cd247* not only exists in T lymphocytes, but also in NK cells. In NK cells, it is associated with activated *NKp46*, *NKp30* and *CD16* (Fc γ RIII) receptors form complexes to achieve signal transduction and subsequent effector functions.^28^ It has been reported that in children with respiratory distress syndrome (RDS), both children with BPD and children without BPD have highly enriched NK “bright” cells,^29^ which may explain the opposite differential expression trend of *Cd247* at the protein level, but its clear mechanism has not been reported yet.

Children with a history of BPD are at a much higher risk of developing the respiratory disease than normal children.^30^, ^31^ Our findings are in agreement with the results reported in the literature and revealed a significant downregulation of TCR signaling, which in turn dysregulates the immune system and increases susceptibility to infections in children.^13^, ^32^, ^33^ Many reports have suggested that T‐cell‐related pathways are associated with postnatal maturation. Numerous studies have also implicated that the pathogenesis of BPD may arise from incomplete development or reduced function of the immune system in preterm infants due to various prenatal risk factors.

This is the first study to explore the crucial pathways and genes associated with BPD via the combination of a BPD animal model with the GEO dataset. The study unveils a pronounced down‐regulation of the T‐cell receptor signaling pathway during the progression of BPD, while a focus on the enriched genes *Cd247*, *Itk*, *Cd3e*, and *Cd3g* related to this pathway provides new insights into the potential etiology of BPD as well as therapeutic targets.

Yet there are some limitations to our study. First, we did not study each time point separately in the course of BPD progression and were unable to compare the pathway and gene expression changes in BPD patients at each time point. Second, our validation of key genes and pathways did not allow an in‐depth understanding of molecular mechanisms. All these limitations warrant further research and exploration.

## AUTHOR CONTRIBUTIONS

Tianyi Wu performed experiments, data analysis and drafted the manuscript. Xingmeng Fu, Xiaoxia Gong, Jingyi You performed experiments and data collection. Zhou Fu performed the guidance of experimental ideas and methods. Chang Shu conceived, designed research and approved the final version of manuscript.

## CONFLICT OF INTEREST STATEMENT

The authors declare that the research was conducted in the absence of any commercial or financial relationships.

## ETHICS STATEMENT

Approval was granted by the Ethics Committee of Children's Hospital of Chongqing Medical University (No. CHCMU‐IACUC20220629003).

## Data Availability

The data that support the findings of this study are openly available in Gene Expression Omnibus at https://www.ncbi.nlm.nih.gov/geo/query/acc.cgi?acc=GSE32472.
